# The impact of chronic obstructive pulmonary disease on surgical outcomes after surgery for an acute abdominal diagnosis

**DOI:** 10.1007/s00068-023-02399-2

**Published:** 2023-12-08

**Authors:** Woubet Tefera Kassahun, Jonas Babel, Matthias Mehdorn

**Affiliations:** Department of Visceral, Transplantation, Thoracic and Vascular Surgery, Faculty of Medicine, University Hospital of Leipzig, Liebigstr. 20, 04103 Leipzig, Germany

**Keywords:** COPD, Acute abdominal diagnosis, Emergency surgery, Postoperative pulmonary complications, Morbidity, In-hospital mortality

## Abstract

**Purpose:**

The current study was undertaken to describe the independent contribution of chronic obstructive pulmonary disease (COPD) to the risk of postoperative morbidity and in-hospital mortality among patients undergoing surgery for an acute abdominal diagnosis.

**Methods:**

Patients who underwent emergency abdominal procedures were identified from the electronic database of the Department of Visceral, Transplantation, Thoracic and Vascular Surgery of our institution. To evaluate differences in surgical risk associated with COPD, patients with COPD were matched for age, sex, and type of surgery with an equal number of controls who did not have COPD. Logistic regression was performed to evaluate the univariate and multivariate associations between the independent variables, including COPD and outcome variables.

**Results:**

Between January 2012 and December 2022, 3519 patients undergoing abdominal emergency surgery were identified in our abdominal surgical department. After removing ineligible cases, 201 COPD cases with an equal number of matched controls remained for analysis. The prevalence of COPD after the exclusion of ineligible cases was 5.7%. There were statistically significant differences in the rate of postoperative pulmonary complications (PPCs [57.7% vs. 35.8%; *P* < 0.001]), ventilator dependence (VD [63.2% vs. 46.3%; *P* < 0.001]), thromboembolic events (TEEs [22.9% vs. 12.9%; *P* = 0.009]), and in-hospital mortality (41.3% vs. 30.8%; *P* = 029) for patients with and without COPD. Independent of other covariates, the presence of COPD was not associated with a significantly increased risk of in-hospital mortality (OR, 1.16; 95% CI 0.70–1.97; *P* = 0.591) but was associated with an increased risk of PPCs (OR, 2.49; 95% CI 1.41–4.14; *P* = 0.002) and VD (OR, 2.26; 95% CI 1.22–4.17; *P* = 0.009).

**Conclusions:**

Preexisting COPD may alter a patient’s risk of PPCs and VD. However, it was not associated with an increased risk of in-hospital mortality.

## Introduction

Abdominal surgery in emergency settings (acute abdomen) is common and addresses a wide range of clinical conditions. It represents up to 11% of all surgical admissions [1]. Due to the life-threatening emergent nature of the underlying condition and resulting inadequate time, preoperative patient optimization is almost impossible. Patients most at risk are those with severe coexisting critical disease [2–4]. Among coexisting conditions, chronic obstructive pulmonary disease (COPD) is characterized by persistent airflow limitations [5]. Its prevalence in the surgical patient population is approximately between 5 and 9% [6, 7]. The importance of COPD as a severe coexisting condition in the pathogenesis, prognosis, and risk of surgery is widely reported. However, the results are conflicting. In some studies, COPD has been recognized as a predictor of poorer early outcomes [8–11], while other studies have failed to clearly establish a link between COPD and increased early morbidity or mortality after surgery [12, 13]. In addition, all those reports mainly included patients after elective surgery, particularly patients after nonemergent coronary artery bypass grafting and abdominal aortic aneurism repair. In contrast, to our knowledge, COPD has not been the subject of a focused study among emergency abdominal surgery patients, despite its potentially serious postoperative consequences.

Therefore, the current study was undertaken to determine the prevalence and independent contribution of COPD to the risk of postoperative morbidity and in-hospital mortality among patients undergoing surgery for an acute abdominal surgical diagnosis. Although relatively infrequent, the situation is one that every abdominal surgeon may confront and deserves reflection.

## Methods

Inclusion criteria

All adult patients with a documented clinical diagnosis of COPD (ICD-10) with or without preoperative spirometry according to their medical records were included. The data of all identified cases were reviewed by one investigator (K.W.T.) to confirm consistency in the diagnosis and complete data.

The exclusion criteria were as follows: patients who reported having COPD but with ambiguous or missing information, patients younger than 18 years of age, patients with a history of asthma, and patients with an active COVID-19 infection.

Institutional ethics committee approval and permission to use the clinical data in an anonymous fashion for scientific reports was obtained for this study. Given the anonymous nature of the data, written informed consent from patients or health care proxies was not required to conduct this research. This study was designed and conducted in adherence to the principles of protection of human subjects set forth in the Declaration of Helsinki. It was written with adherence to the Strengthening the Results of Observational Studies in Epidemiology (STROBE) [14] statement.

We included a series of 3519 consecutive patients who underwent emergency abdominal procedures at the Department of Visceral, Transplantation, Thoracic and Vascular Surgery of the University of Leipzig. These patients were evaluated for inclusion based on the inclusion criteria. Data were collected from the electronic database of our surgical department. Emergency abdominal operations were defined as any surgery that had to be performed as soon as possible after admission or after the onset of related clinical symptoms.

The records of patients who underwent operations for an acute abdominal surgical diagnosis from January 2012 to December 2022 were retrospectively reviewed to establish the prevalence of COPD. COPD was defined as any outpatient physician visit or hospital admission in which COPD was recorded as a diagnosis regardless of its duration according to the International Statistical Classification of Diseases and Related Health Problems, tenth revision [ICD-10; J44.80, J44.81, J44.82, J44.83, J44.89, and J44.90].

Patients for this study were derived partly from our previous study, in which we analyzed the effects of reoperation on surgical outcomes [15]; however, this study focused on patients with preexisting COPD who required emergency surgery.

Patient demographics and clinical variables were prospectively collected. Discharge dispositions and in-hospital deaths were recorded.

Data for patients with and without (reference group) COPD were analyzed separately to assess the effects of COPD on postoperative morbidity and in-hospital mortality following emergency abdominal surgery for an acute abdominal surgical diagnosis. After excluding cases (COPD group), 201 controls (non-COPD group) were randomly selected from among patients undergoing emergency surgery for an acute abdomen based on matching criteria (same age, same sex and same procedure). The charts of the control patients were reviewed for the same factors as the charts of the case patients, as appropriate. The matched pair characteristics (those variables that were used in the matching algorithm) were based on collected data from the electronic database, which captures all accessible electronic data throughout the hospital database system. According to this algorithm, the COPD and non-COPD patients were similar. Both groups were sicker upon admission than the entire emergency cohort, and the patients with COPD were even sicker than the patients without COPD. Regarding the comorbidity burden, we tried to match cases and controls. However, the revision of all available charts resulted in either the case missing its control or the control missing its case.

Because surgery was performed emergently, preoperative pulmonary function testing was not performed on a routine basis. Therefore, the severity of COPD defined by pulmonary function tests was not determined in this study.

In 112 patients (55.7%), spirometric findings (forced vital capacity [FVC], forced expiratory volume in 1 s [FEV1]) compatible with a COPD diagnosis [16] were reported. According to those findings, this subgroup of patients was divided as follows: Mild to moderate COPD (FEV1/FVC ratio < 70%, FEV1 ≥ 50% of predicted value), *n* = 57 (50.9%) and Severe COPD (FEV1/FVC ratio < 70%, FEV1 < 50% of predicted value), *n* = 55 (49.1%).

Due to the underutilization of spirometry in community practice, the diagnosis of COPD may rely solely on clinical grounds for a large number of patients. According to the study by Han et al. [17], only 32% of a broad range of patients with a new COPD diagnosis had undergone spirometry within the previous 2 years–6 months following diagnosis. In another study, Joo et al. [18] confirmed the observations made by those authors. According to their results, only 36.7% of patients with new COPD underwent spirometry. In our study, the majority of studied COPD patients (55.7%) had spirometric findings. However, for 89 (44.3%) patients, no spirometric findings were available. In these patients, the diagnosis of COPD as a coexisting disease was clinical and established by reviewing the available documents on the patient´s entire medical history, including any treatment for the condition.

The severity of medical conditions at the time of surgery was evaluated using the American Society of Anesthesiologists (ASA) physical status classification [19]. The Clavien‒Dindo classification (CDC) of surgical complications [20] was used to classify surgical complications. In addition, based on the CDC grade at discharge, the comprehensive complication index (CCI) [21] was calculated for each patient to evaluate the true overall morbidity burden of a procedure.

The outcome measures were 1. in-hospital mortality defined as in-hospital death from any cause during the same hospitalization as the surgical procedure; 2. the occurrence of overall postoperative complications; 3. postoperative pulmonary complications (PPCs), which was defined as the occurrence of one or more of the following: postoperative pneumonia, postoperative respiratory failure, atelectasis, and acute respiratory distress syndrome. Although the clinical significance of each of these complications varies, they have all been classified as PPCs and often grouped together in studies on risk factors for PPCs [22, 23]; 4. prolonged noninvasive or invasive mechanical ventilation (PMV), defined as the need for mechanical ventilation beyond the operation room after completion of the operation; and 5. length of stay in the hospital and ICU.

Data for patients with and without COPD were analyzed separately to assess the effects of COPD on outcome measures. Logistic regression was performed to evaluate the univariate and multivariate associations between the independent variables, including COPD and outcome variables. In univariate analysis, continuous and categorical variables were compared using a two-tailed Student´s t test and Pearson´s chi-square test, respectively. Multivariable logistic regression analysis assessed risk factors for negative outcomes. Forward selection within the regression model was stepwise, alternating between dropping the least significant variable from the model and then reconsidering all potential variables for reintroduction into the model until no more variables could be added. The entry and exit criteria were set at P ≤ 0.1 and P ≤ 0.05, respectively. Odds ratios (ORs) and confidence intervals (CIs) were reported for the logistic regression model. Differences were considered significant at p ≤ 0.05. SPSS software package version 29 for Windows (IBM Corporation, USA) was used to perform the statistical analysis.

## Results

**Fig. 1:**
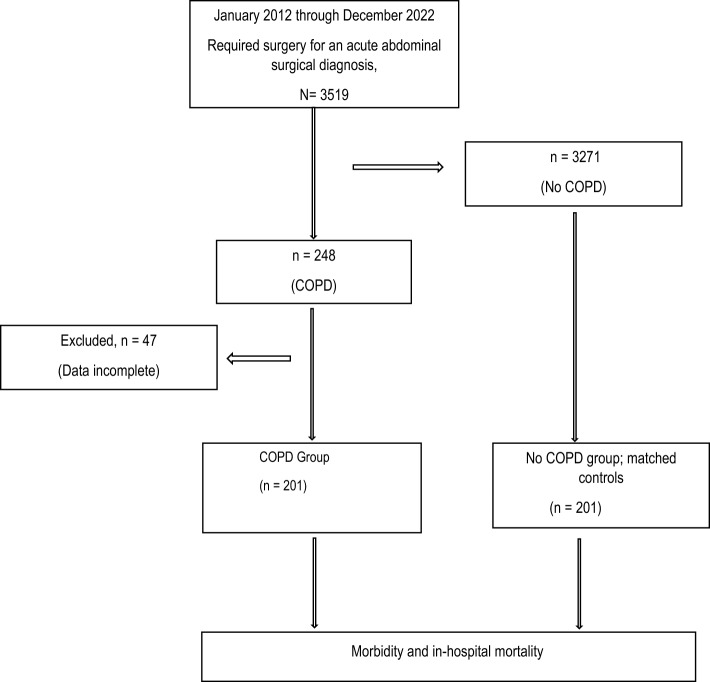
Flow diagram of patient selection

Between January 2012 and December 2022, 3519 patients undergoing abdominal emergency surgery were identified in our abdominal surgical department. We reviewed all those cases. Of those patients, 248 patients with COPD required abdominal surgery for an acute abdominal surgical diagnosis. After removing 47 ineligible cases, we were left with 201 COPD cases for analysis. To evaluate differences in surgical risk associated with COPD, the 201 patients with COPD were matched for age, sex, and type of surgery with an equal number of controls who did not have COPD taken from a cohort of 3271 emergency abdominal surgical patients without preexisting COPD. Hence, the final sample consisted of 201 cases (COPD group) and 201 matched controls (non-COPD group) available for analysis (Fig. 1).

The prevalence of COPD in the entire cohort of abdominal emergency patients was 5.7%. Preoperative and demographic variables as well as the relative frequencies of the coexisting conditions in both groups are detailed in Table 1. Overall, 63% of all emergency abdominal surgical patients were male, and 37% were female. The sex distribution and the mean age were similar among patients with and without COPD after matching. Based on the frequency of coexisting conditions (COCs) and ASA class, both groups had more chronic illnesses than the entire emergency population.

**Table 1:** Baseline patient characteristics

	All patients	COPD	No COPD	P* value
Variable	*N* = 402	*N* = 201	*N* = 201	
Male	254 (63.2)	127 (63.2)	127 (63.2)	1.000
Age, years, mean ± SD	69.60 ± 12.01	69.63 ± 12.14	69.57 ± 11.91	0.960
Age, years, ≥ 70	204 (50.7)	101 (50.2)	103 (51.2)	0.921
BMI, mean ± SD	26.90 ± 6.66	26.96 ± 6.50	26.83 ± 6.83	0.846
Comorbid conditions (COCs)	90 (97.0)	197 (98.0)	193 (96.0)	0.241
ASA^a^ ≥ 3	316 (80.6)	174 (87.9)	142 (73.2)	< 0.001
Major COCs				
Hypertension	299 (74.4)	160 (79.6)	139 (69.2)	0.016
AF	135 (33.6)	81 (40.3)	54 (26.9)	0.004
CHF	99 (24.6)	67 (33.3)	32 (15.9)	< 0.001
PAD	114 (28.4)	67 (33.3)	47 (23.4)	0.027
Diabetes mellitus	114 (28.4)	64 (31.8)	50 (24.9)	0.121
IHD	93 (23.1)	60 (29.9)	33 (16.4)	0.001
CKD	98 (24.4)	60 (29.9)	38 (18.9)	0.011
Liver cirrhosis	30 (7.5)	12 (6.0)	18 (9.0)	0.255
History of malignant disease	138 (34.3)	68 (33.8)	70 (34.8)	0.834

*COPD* chronic obstructive pulmonary disease, *BMI* body mass index in kg/m^2^, *SD* standard deviation, *AF* atrial fibrillation, *CHF* congestive heart failure, *PAD* peripheral arterial disease, *CKD* chronic kidney disease, *COCs* comorbid conditions excluding COPD, *ASA* the American society of anesthesiologists physical status classification

Numbers in bracket indicate values presented in *n* (%) by group unless noted otherwise

*P values represent the difference between groups with and without preexisting COPD

^a^Percents may not total 100 due to missing data

Among the coexisting conditions, hypertension (79.6%) was the most frequent, followed by atrial fibrillation (AF [40.3%]), congestive heart failure (CHF [33.3%]), peripheral arterial disease (PAD [33.3%]), diabetes (31.8%), chronic kidney disease (CKD [29.9%]), and ischemic heart disease (IHD [29.9%] in the COPD group. The rank order of coexisting conditions was virtually identical by group except for diabetes and PAD, which were the 3rd and 4th most frequent coexisting conditions in the non-COPD group.

Table 2 lists the primary indications for emergency abdominal surgery and index emergency procedures, including surgical access (laparoscopy vs. open). As presented in this table, indications for emergency surgery were relatively balanced without significant differences, and all index procedures were virtually identical between the COPD and non-COPD groups.

**Table 2:** Primary Indications for emergency surgery and index procedures by group

	All patients	COPD	No COPD
	*N* = 402	*N* = 201	*N* = 201
Surgical emergency			
Viscous organ perforation	120 (29.9)	52 (25.9)	68 (33.8)
Mesenteric ischemia	78 (19.4)	45 (22.4)	33 (16.4)
Bowel obstruction	74 (18.4)	38 (18.9)	36 (17.9)
Cholecystitis	47 (11.7)	24 (11.9)	23 (11.4)
Appendicitis	30 (7.5)	15 (7.5)	15 (7.5)
Hemorrhage	15 (3.7)	6 (3.0)	9 (4.5)
Miscellaneous	38 (9.5)	21 (10.5)	17 (8.5)
Index procedure			
Small bowel resection	42 (10.5)	21 (10.5)	21 (10.5)
Hartmann’s procedure	44 (10.9)	22 (10.9)	22 (10.9)
Right colectomy	40 (10.0)	20 (10.0)	20 (10.0)
Colon resection unspecified	4 (1.0)	2 (1.0)	2 (1.0)
Closure of viscus organ	48 (11.9)	24 (11.9)	24 (11.9)
Control of hemorrhage	12 (3.0)	6 (3.0)	6 (3.0)
Simultaneous MPs	34 (8.5)	17 (8.5)	17 (8.5)
Lysis of adhesions	78 (19.4)	39 (19.4)	39 (19.4)
Cholecystectomy	38 (9.5)	19 (9.5)	19 (9.5)
Appendectomy	30 (7.5)	15 (7.5)	15 (7.5)
Laparotomy only	14 (3.5)	7 (3.5)	7 (3.5)
Repair of intestinal perforation with formation of stoma	10 (2.5)	5 (2.5)	5 (2.5)
Miscellaneous procedures	8 (2.0)	4 (2.0)	4 (2.0)
Access to abdomen			
Laparoscopy	116 (28.9)*	58 (28.9)*	58 (28.9)*
Conversion	40 (34.5)	20 (34.5)	20 (34.5)
Laparotomy	326 (81.1)^a^	163 (81.1)^a^	163 (81.1)^≠^

Numbers in bracket indicate values presented in *N* (%)

*MPs* multiple procedures

*Indicates all initially attempted laparoscopies without exclusion of converted patients

^a^All open procedures including converted cases

Viscous organ perforation was the most common indication for emergency surgery (29.9%), followed by mesenteric ischemia (19.4%) and bowel obstruction (18.4%).

Bowel resection was the most common procedure performed, accounting for 32.6% of all procedures. Extensive lysis of adhesions was the second most common procedure, accounting for 19.4% of procedures, and closure of viscous organs for 11.9% of procedures. Excluding conversion from laparoscopy to open surgery, 38 patients in each group underwent laparoscopic procedures. These procedures were mainly appendectomies and cholecystectomies.

Table 3 displays the percentage of outcome variables in the groups with and without COPD along with the *P* values from the chi-square test for categorical variables and Student´s *t* test for continuous variables. While most patients in the entire emergency cohort presented a single postoperative complication (67.7%; data not shown), 62.3% of COPD patients and 55.5% of controls experienced 2 or more complications. However, the difference between cases and controls regarding the occurrence of postoperative complications overall was not significant. Similarly, the proportion of patients requiring an ICU stay was higher in COPD patients than in controls (74.1% vs. 63.2%), and this difference did reach statistical significance at *P* = 0.030. Of the 201 patients with preexisting COPD, 83 died, for an in-hospital mortality rate of 41.3%, followed by those who underwent emergency abdominal operations with 62 deaths, for an in-hospital mortality rate of 30.8%, in the control group of patients without COPD. This difference was statistically significant at *P* = 0.029. Furthermore, there were statistically significant differences in the rate of PPCs (57.7% vs. 35.8%; *P* < 0.001]), ventilator dependence (VD [63.2% vs. 46.3%; *P* < 0.001]), and thromboembolic events (TEEs [22.9% vs. 12.9%; *P* = 0.009]) that included deep venous thrombosis, pulmonary embolism, myocardial infarction, ischemic stroke and systemic embolism for patients with and without COPD. No statistically significant differences were observed for other outcome variables.

**Table 3:** Overall outcomes between patients with and without preexisting COPD

	All patients	COPD	No COPD	P*
Variable	*N* = 402	*N* = 201	*N* = 201	
Complications overall	273 (67.9)	145 (72.1)	128 (63.7)	0.069
Multiple complications	230 (57.2)	125 (62.2)	105 (52.2)	0.088
Cpp, mean ± SD	5.27 ± 3.27	5.19 ± 3.18	5.37 ± 3.40	0.647
CCI mean ± SD	56.8 ± 37.25	60.51 ± 37.20	53.06 ± 37.03	0.045
PPCs	188 (46.8)	116 (57.7)	72 (35.8)	< 0.001
Pneumonia	103 (25.6)	60 (29.9)	43 (21.4)	0.052
Bleeding events	87 (21.6)	44 (21.9)	43 (21.4)	0.904
TBP	102 (25.4)	47 (23.4)	55 (27.4)	0.359
SSI	141 (35.1)	65 (32.3)	76 (37.8)	0.250
Anastomotic leak	50 (12.4)	22 (10.9)	28 (13.9)	0.365
TEEs	72 (17.9)	46 (22.9)	26 (12.9)	0.009
Liver failure	101 (25.1)	54 (26.9)	47 (23.4)	0.421
ARF	168 (41.8)	89 (44.3)	79 (39.3)	0.312
URLs	148 (36.8)	80 (39.8)	68 (33.8)	0.215
ICU	276 (68.7)	149 (74.1)	127 (63.2)	0.030
ICU-LOS, days, median	7 (1–127)	7 (1–127)	6 (1–78)	0.431
VD	220 (54.7)	127 (63.2)	93 (46.3)	< 0.001
DMV, hours, median	61 (1–1825)	62 (2–1641)	60 (1–1825)	0.504
LOS, days, median	15 (1–154)	16 (1–154)	15 (1–90)	0.660
In-hospital mortality	145 (36.1)	83 (41.3)	62 (30.8)	0.029

*COCs* comorbid conditions, *Cpp* complications per patient, CCI the comprehensive complication index, *PPCs* postoperative pulmonary complications, *SSI* surgical site infection is defined as being contained within the skin or subcutaneous tissue (superficial) or involving the muscle and /or fascia (deep), *TEEs* thromboembolic events, *ARF* acute renal failure that required dialysis, *URLs* unplanned relaparotomies, *VD* ventilator dependence, *ICU* intensive care unit, *LOS* hospital length of stay

*p values represent the difference between groups with and without COPD

In a further univariate analysis, we determined the odds of mortality for a variety of factors, including COPD. As shown in Table 4, each of the 18 risk factors, including COPD, was a significant univariate predictor of in-hospital mortality.

**Table 4:** Odds of mortality for all patients (COPD and CONTROLS, *N* = 402; univariate analysis)

Variable	OR (95% CI)	*P*-value
COPD	1.56 (1.05–2.38)	0.029
Age ≥ 70 years	1.88 (1.24–2.84)	0.003
ASA ≥ 3	18.62 (5.75–60.36)	< 0.001
AF	2.17 (1.42–3.33)	< 0.001
CHF	1.51 (0.95–2.41)	0.079
PAD	3.64 (2.31–5.72)	< 0.001
Liver cirrhosis	2.89 (1.35–6.20)	0.005
PPCs	29.74 (16.17–54.69)	< 0.001
Pneumonia	6.34 (3.88–10.34)	< 0.001
ICU	9.87 (5.09–19.13)	< 0.001
Bleeding events	4.90 (2.96–8.13)	< 0.001
BPT	6.56 (4.01–10.75)	< 0.001
TEEs	7.23 (4.05–12.85)	< 0.001
Anastomotic leak	2.33 (1.28–4.24)	0.005
ARF	40.85 (22.24–75.03)	< 0.001
Liver failure	79.48 (32.40–191.45)	< 0.001
VD	18.92 (11.17–32.07)	< 0.001
URLs	3.95 (2.57–6.10)	< 0.001

*OR* odds ratio, *CI* confidence interval, *ASA* the American society of anesthesiologists physical status classification, *AF* atrial fibrillation, *VD* ventilator dependence, *PAD* peripheral arterial disease, *IHD* ischemic heart disease, *CKD* chronic kidney disease, *COPD* chronic obstructive pulmonary disease, *TEEs* thromboembolic events, ARF acute renal failure that required dialysis, *BPT* blood product transfusion, *ICU* intensive care unit stay, *URLs* unplanned relaparotomies

Following univariate analysis, we performed multivariable stepwise logistic regression analysis for negative outcomes. We analyzed COPD as an independent risk factor for each of the following individual postoperative events as the outcome of interest separately: in-hospital mortality, PPCs, VD, and length of stay (LOS) in the ICU and hospital. The analysis showed the independent effect of COPD after adjusting for a variety of patient and disease characteristics, as represented in the table legends of Tables 5 and 6. We present the adjusted ORs, their 95% CIs, and P values. Independent of other covariates, the presence of COPD was not associated with a significantly increased risk of in-hospital mortality (OR, 1.16; 95% CI 0.70–1.97; *P* = 0.591) but was associated with an increased risk of PPCs (OR, 2.49; 95% CI 1.41–4.14; *P* = 0.002) and VD (OR, 2.26; 95% CI 1.22–4.17; *P* = 0.009). Furthermore, there was no significant association between COPD and an increased risk of complications overall or LOS. Variables with a significant association with in-hospital mortality were PPCs (OR, 5.04; 95% CI 2.17–11.71; *P* =  < 0.001), ARF (OR, 3.65; 95% CI 1.51–8.84), LF (OR, 13.24; 95% CI 4.81–36.43; *P* =  < 0.001) and VD (OR, 2.58; 95% CI 1.15–5.81; *P* = 022). Regarding in-hospital death, the multivariate regression analysis reduced the predictor variables to those that were most unique and discriminating.

**Table 5:** Multivariate analysis (COPD and Controls, *N* = 402): effect of COPD on outcome variables after adjusting for risk factors

Outcome variable	OR (95% CI)	*P*
PPCs	2.49 (1.41–4.41)	0.002
VD	2.26 (1.22–4.17)	0.009

*PPCS* postoperative pulmonary complications overall, *URLs* unplanned reoperations, *ICUS* intensive care unit length of stay, *VD* ventilator dependence, *LOS* hospital length of stay

Adjusted for COPD, Age ≥ 70 years, Sex, BMI ≥ 30 kg/m^2^, ASA ≥ 3, AF, IHD, CHF, PAD, CKD, liver cirrhosis, Hypertension, Diabetes mellitus, and URLs

**Table 6:** Multivariate analysis (COPD and Controls, *N* = 402): predictors of in-hospital mortality after adjusting for risk factors

Risk factor	OR (95% CI)	*P* value
PPCs	5.04 (2.17–11.71)	< 0.001
ARF	5.0 (2.33–10.75)	< 0.001
Liver failure	13.24 (4.81–36.43)	< 0.001
VD	2.58 (1.15–5.81)	0.022

*PPCS* postoperative pulmonary complications overall, *VD* ventilator dependence, *LOS* hospital length of stay, *ARF* acute renal failure that required dialysis

Adjusted for COPD, Age ≥ 70 years, Sex, ASA ≥ 3, AF, IHD, CHF, PAD, CKD, PPCs, liver cirrhosis, VD, ICU stay, Pneumonia, bleeding events, BPT, anastomotic leaks, TEEs, liver failure, ARF, URLs, and ICU. Each of the risk factors included in the model represents a significant univariate predictor of in-hospital mortality (Table 4)

## Discussion

The present study was undertaken to determine the impact of preexisting COPD on outcomes after surgery for an acute abdominal surgical diagnosis. The prevalence of COPD in our entire cohort of emergency patients was 5.7%. This agrees with previous research that found a comparable prevalence of COPD among surgical patients [7].

The results suggest that compared to controls, patients with preexisting COPD had an increased postoperative pulmonary complication rate, ICU admission rate, ventilator dependence rate and in-hospital death rate after emergency abdominal surgery. No statistically significant differences were observed in the overall complication rate or for prolonged LOS in the ICU or hospital. Furthermore, multivariable regression analysis showed that preexisting COPD was associated with an increased risk of PPCs and VD. There was no significant association between COPD and in-hospital mortality.

To our knowledge, the current study is the first to report on the outcomes of a sizable number of patients with preexisting COPD compared with matched controls after emergency abdominal surgery.

Almost 72% of these patients had at least 1 postoperative complication, and 41% died in the hospital. This implies that, as reported by others [6] regarding elective surgery, preexisting COPD affects the outcomes of the abdominal emergency patient population as well.

The observed incidence of a composite outcome of PPCs, the rate of ventilator dependence and in-hospital mortality were higher than previous outcome reports despite high-level hospital services, including ICU care. This is likely due to a combination of different patient characteristics and high-risk emergency surgical procedures. For example, 88% of COPD patients had an ASA class equal to or above 3 with severe systemic disease, and COPD patients had on average 6 coexisting conditions per patient. Patients with severe coexisting conditions on admission are more than seven times more likely to have a complication than those without such conditions [24].

Therefore, in addition to the complexity of disease and surgical treatment in emergencies, where the acute insult is greater, many preexisting factors increase the risk that a patient will have an adverse event following surgery during hospitalization.

To our knowledge, these findings have not been reported with regard to the outcomes of COPD patients after surgery for emergency abdominal diagnosis in a large patient cohort (cases and controls), and relevant data are scarce.

The majority of previous studies concentrated mainly on the outcomes of COPD patients after elective surgery [5, 9, 10, 12, 25, 26]. In those studies, the included number of emergency cases of COPD patients was either unknown or very small without matched controls, making it difficult to draw a conclusion from the analysis. Thus, the overall information generated by those studies regarding the effect of COPD on outcomes after surgery in the emergency setting was inconclusive.

Patient characteristics (patient factors) also increased the risk of an adverse event following surgery. For example, 51% of the studied patients were older than or aged 70 years, and elderly patients had more complicated disease that increased the risk of complications and in-hospital mortality after surgery [27–31].

Interestingly, these outcomes did not vary significantly with the severity of COPD. For example, our analysis of the subgroup of patients with valid spirometric results showed that 65% of the patients with mild to moderate COPD and 76% with severe COPD suffered at least 1 postoperative complication. Similarly, the in-hospital mortality rates were 38.6% and 36.4% among patients with mild to moderate and severe COPD, respectively (data not shown). This is in agreement with the findings of the study by Manganas et al. [12] that reported on outcomes after elective coronary artery bypass grafting. These authors found no association between the severity of airflow obstruction and the mortality rate. In another study among patients after elective abdominal surgery, Kim et al. [5] found no association between mild-to-moderate COPD and the risk of PPCs. However, because of the small sample size for each spirometric stage in our study, as described in detail in the Methods section, comparison of the obtained findings was difficult, and the results must be interpreted with caution.

In the current study, COPD was independently associated with an increased risk of PPCs and VD but not in-hospital mortality. This is in agreement with previous reports that suggested that COPD is an independent risk factor for PPCs and VD but not for in-hospital mortality after controlling for other covariates [8, 12, 25, 32].

Of the complications that we included for analysis, PPCs were the most common after emergency abdominal surgery and had a strong association with in-hospital mortality. Other index complications were less common. Therefore, according to the results of our study, preexisting COPD may predispose patients to mortality indirectly through its impact on PPCs following surgery for abdominal emergencies. The lack of a clear association between COPD and in-hospital mortality following surgery for abdominal emergencies may reflect the dominance of other factors, such as pulmonary complications and acute multiple organ failure, which were all noted in previous studies [15, 33, 34], in determining the clinical course of these patients. In general, it is our impression that the impact of COPD on mortality was offset by the dominance of other factors.

For patients undergoing elective surgery, preexisting comorbid conditions, including COPD, can be modified to reduce the risk of severe complications and improve the outcome. Unfortunately, in unforeseen and life-threatening emergency events, preoperative risk factor modification is almost impossible. On the other hand, surgery is mandatory because almost all of these patients who had emergency operations would have died of the condition for which they were treated if left untreated. Therefore, while treating patients with preexisting COPD for an abdominal emergency where the acute insult is greater, surgeons should be aware of expected severe and multiple complications, including prolonged ventilator dependence. More personal attention and information regarding high-risk procedures, personal experiences and previous outcomes should be integrated into the treatment and communicated with the patient (if possible), the families and health care proxies. Efforts to improve the postoperative outcomes of such patients will necessarily require a focus on the prevention of postoperative morbidity and on the timely diagnosis and management of complications that do occur, as suggested by others [28, 35, 36].

Several limitations of this study merit attention. First, the current study was limited by its retrospective nature. As such, potential bias may have been introduced as a result of systemic coding errors, lack of accurate severity adjustment for comorbid conditions, and absence of adequate information on health-related behaviors, particularly smoking, as a central risk factor for COPD. Our data collection relied on existing documentation that was not specifically collected for this study or its variables. Furthermore, this study involved patients from a single center and was not designed to attribute causality between preexisting COPD and poor surgical outcomes.

Second, the present study was limited by the inability to stratify patients by COPD severity. Not all patients had a valid spirometric finding. For some patients, we were unable to determine whether missing spirometric data were absent because the associated findings were truly absent or whether the findings were present but not recorded in our electronic database. However, the impact of this limitation was likely to be small because, as discussed above, the postoperative mortality and morbidity among those patients with valid spirometric results were similar to those of patients without.

Third, the COPD group was sicker (higher comorbidity burden) than the non-COPD group. However, in the multivariable analysis, we adjusted for all of these comorbid conditions to predict outcomes based on adjusted variables. Therefore, once a multivariable analysis was completed, it would have adjusted for the difference.

Fourth, it is possible that the associations between COPD and the outcomes we described were impacted by other variables that we might not have accounted for.

Despite these limitations, our analysis of a relatively large cohort of matched patients undergoing surgery for an acute abdominal surgical diagnosis demonstrated preexisting COPD to be an important contributor to subsequent postoperative outcomes. This study represents a real-life situation in which surgeons are confronted with COPD patients who need surgery as soon as possible during the same admission in which the diagnosis was made. Because of the relatively large sample size, the results of this study more closely approximate the true impact of COPD on outcomes after emergency abdominal surgery than results from studies with smaller cohorts of patients.

## Conclusions

Overall, our study demonstrated the impact that preexisting COPD had on outcomes after surgery for an acute abdominal diagnosis. There was an association between COPD and PPCs/VD but not in-hospital mortality after emergency surgery. The association of preexisting COPD with the reported outcomes emphasizes its clinical importance. In this regard, interventions to reduce postoperative PPCs as well as their timely diagnosis and focused treatment when they occur may offer opportunities for improvements in outcomes.
